# High-order synchronization of hair cell bundles

**DOI:** 10.1038/srep39116

**Published:** 2016-12-15

**Authors:** Michael Levy, Adrian Molzon, Jae-Hyun Lee, Ji-wook Kim, Jinwoo Cheon, Dolores Bozovic

**Affiliations:** 1 Department of Physics and Astronomy, California NanoSystems Institute, University of California, Los Angeles, California 90095, United States; 2 Center for Nanomedicine, Institute for Basic Science (IBS), Seoul 03722, Republic of Korea; 3 Yonsei-IBS Institute, Yonsei University, Seoul 03722, Republic of Korea; 4 Department of Chemistry, Yonsei University, Seoul 03722, Republic of Korea

## Abstract

Auditory and vestibular hair cell bundles exhibit active mechanical oscillations at natural frequencies that are typically lower than the detection range of the corresponding end organs. We explore how these noisy nonlinear oscillators mode-lock to frequencies higher than their internal clocks. A nanomagnetic technique is used to stimulate the bundles without an imposed mechanical load. The evoked response shows regimes of high-order mode-locking. Exploring a broad range of stimulus frequencies and intensities, we observe regions of high-order synchronization, analogous to Arnold Tongues in dynamical systems literature. Significant areas of overlap occur between synchronization regimes, with the bundle intermittently flickering between different winding numbers. We demonstrate how an ensemble of these noisy spontaneous oscillators could be entrained to efficiently detect signals significantly above the characteristic frequencies of the individual cells.

Synchronization and entrainment phenomena have been shown to be ubiquitous in nonlinear oscillators. Already in the 17^th^ century, Huygens demonstrated that two clocks, weakly coupled by a wall, synchronize the swings of their pendula^1^. Synchronization was investigated in many nonlinear mechanical and electrical devices, from musical instruments to lasers. Mathematical studies have extensively explored the entrainment of a nonlinear oscillator by an imposed oscillatory drive^2^,^3^,^4^. In recent years, a number of biological systems were shown to exhibit nonlinearities in their response to external signals and to display synchronization between constituent oscillators^2^,^5^,^6^,^7^,^8^,^9^,^10^,^11^. For example, synchronization was encountered in collective animal behaviors^7^,^8^, in cooperation between organs^9^,^12^, or even at the level of individual cells^10^,^11^. Investigating synchronization and entrainment is of particular interest in explaining the complex nonlinear dynamics of the auditory system. For the sense of hearing, a phase-locked mechanical response to an external sound constitutes a crucial step in auditory detection. Entrainment occurs at many levels in the process of hearing, starting with the response of mechanosensitive hair cells^13^.

Hair cells of the inner ear are biological sensors that detect displacements induced by air- or ground-borne vibrations and transduce them into electrical signals^14^,^15^. They are composed of a cell body and a bundle of 30–50 stereocilia that protrude from the apical surface^15^,^16^. Near the thresholds of detection, these cells can be responsive to bundle motions as small as a few Å^17^. Furthermore, the auditory system is sensitive to a broad range of frequencies, with some species able to detect signals up to 100 kHz and higher^18^. The biophysical mechanisms behind the extreme sensitivity of detection and the ability to respond to mechanical signals at such high frequencies are still not fully understood.

A compressive nonlinearity arises in the evoked response, measured both in the motility of individual hair bundles and in the response of the whole system - vibrations of the membrane in which the cells are embedded^15^,^17^,^19^. The nonlinear response was demonstrated in a number of different species^15^,^20^,^21^,^22^, and was shown to be crucial to the sensitivity of detection by the hair cells. Further, it was shown to be an essential nonlinearity^23^, which is maintained even as incoming signals approach zero amplitude.

Spontaneous oscillations of hair bundles have been observed in several species^24^,^25^,^26^. They result from an internal amplifier that enables the hair cells to sustain their sensitivity under over-damped *in vivo* conditions^15^,^21^,^27^,^28^. Two cellular processes are involved in the oscillation process. Gating of the mechanically sensitive ion channels in the stereocilia leads to bi-stability in the position of the bundle. An array of myosin motors has been proposed to be physically connected to the transduction complex, and to climb and slip along the actin filaments that form the core of the stereocilia. This adaptation process continuously adjusts the position of the bundle and, in conjunction with mechanical gating of the transduction channels, leads to spontaneous oscillations^26^. The characteristic frequency of these spontaneous oscillations defines an internal time scale for the mechanical response of the bundle. Surprisingly, this natural frequency is lower than the frequency range of detection of the corresponding organs. For example, the frog sacculus can detect frequencies up to 120 Hz^29^, while spontaneous oscillations of individual bundles *in vitro* typically display characteristic frequencies around 30 Hz^26^. In the current study, we assess whether an ensemble of slow nonlinear oscillators could efficiently encode frequencies higher than those of their internal clocks. We describe experiments that measure the mechanical response of the sensory epithelium to frequencies covering the full detection range.

One of the common features of nonlinear oscillators is that they can support multi-mode phase-locking^4^. Systems that are exposed to varying frequencies and amplitudes of stimulus can phase-lock to the imposed signal in a variety of synchronization modes, forming regions of entrainment referred to as Arnold Tongues. We explore whether hair cells of the inner ear exhibit multi-mode synchronization and how this mode-locking could affect higher frequency detection.

A recently developed technique, which takes advantage of nanomagnetism to stimulate hair cells, is used to probe the bundle response at higher frequencies. We observe regimes of synchronization corresponding to different mode-locking ratios. Unlike traditional Arnold Tongues encountered in dynamical systems literature, the measured phase-locking regions show overlapping patterns, with hair bundles intermittently switching between different high-order modes. Numerical simulations suggest potential implications of such behavior for the detection of frequencies higher than the natural frequencies of individual bundles.

## Results

### Magnetic stimulation

To study the mechanical response of an individual hair bundle, one must apply deflections that mimic those induced by incoming sounds under *in vivo* conditions. Traditionally, these deflections were imposed either with elastic glass rods, or with fluid jets. To access higher frequencies of stimulation without imposing a mechanical load on the bundles or introducing hydrodynamic artifacts, we used an electromagnetic probe to attract superparamagnetic beads that are conjugated to hair bundles. The beads were composed of iron oxide (Fe_3_O_4_) nanoparticles embedded in a 1 μm polymer structure. Their superparamagnetic behavior leads to an instantaneous cooperative action of all the nanoparticles, and hence allows one to impose a substantial force to deflect the bundles. This novel technique was recently demonstrated to enable bundle stimulation at kHz frequencies^30^, well above the requirements of the current study.

Experiments were performed at room temperature on saccular hair cells of the North American bullfrog (*Rana catesbeiana*). The sacculi were excised from the inner ear and mounted over a 1 mm hole in a two-compartment experimental chamber, with hair cells exposed to artificial perilymph on the basal side and endolymph on the apical side, as described in previous publications^30^,^31^ (see Materials and Methods).

**Figure 1:**
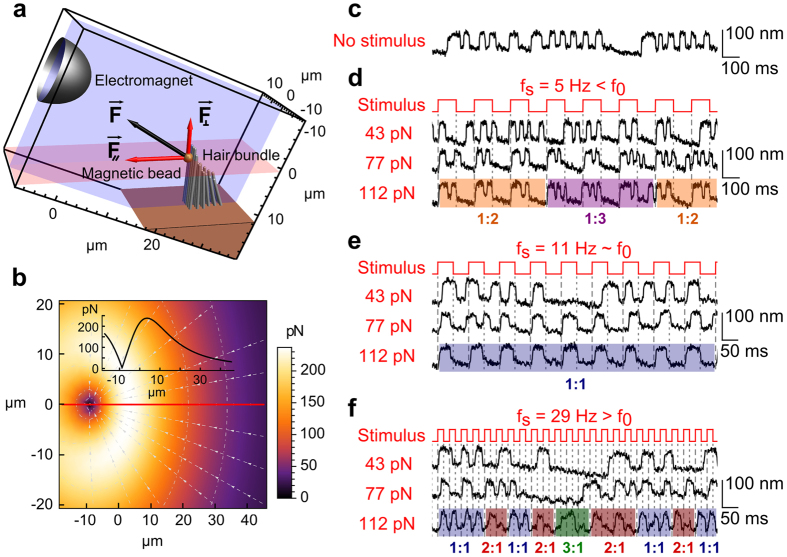
Experimental setup and entrainment of hair bundle dynamics. (**a**) Scaled schematic of a hair bundle that protrudes from the apical surface of the saccular epithelium; the bundle is coupled to a magnetic bead and stimulated by the electromagnetic probe. The symmetry plane and the plane of bundle motility are indicated in blue and red respectively. (**b**) Projection of the magnetic force acting on an average bead onto the plane of bundle motility (red plane). The origin of the plane corresponds to the projection of the tip onto the plane. The inset presents the force intensity along the red line. (**c**) Spontaneous oscillations of a hair bundle. Hair bundle oscillations in the presence of a square-wave stimulus, applied at increasing intensities for (**d**) *f*_*s*_ < *f*_0_, (**e**) *f*_*s*_ ~ *f*_0_, and (**f**) *f*_*s*_ > *f*_0_. The modes of synchronization are indicated for the highest stimulation intensity.

The magnetic beads were conjugated to the hair bundles^32^ (see Materials and Methods), and an electromagnetic probe, positioned in close proximity (~10–20 μm) to a target hair bundle, was used to impose a square wave stimulus, at various frequencies and force intensities. The nanoparticles being superparamagnetic, they could only be pulled by the electromagnet and not pushed. As a consequence, a rectangular stimulus was the most natural choice. The magnetic force generated by the probe tip on an average bead was calibrated, as shown in Fig. 1a and b. Spontaneous and evoked bundle motility was recorded with a CMOS camera.

### Phase-locking of oscillating bundles

Figure 1c,d,e, and f show examples of the innate motility exhibited by a hair bundle and its entrainment by stimulation applied at frequencies that are lower, comparable, or higher than the characteristic frequency *f*_0_ of the bundle. For a stimulus frequency *f*_*s*_ close to *f*_0_, the spontaneous oscillation exhibits progressively stronger 1:1 phase-locking with increasing intensity of the drive. However, a stimulus applied at a frequency that is significantly different from *f*_0_ evokes a more complex behavior, with different modes of phase synchronization dominating *alternately*. The natural frequency *f*_0_ was evaluated for each bundle from a ten second recording of spontaneous oscillations.

### Synchronization pattern

A nonlinear oscillator that exhibits multi-mode phase locking can form Arnold Tongues. To analyze the patterns of mode-locking between an autonomously oscillating bundle and an imposed drive, we followed mathematical procedures developed for the study of entrainment in noisy and/or chaotic systems. The usual condition for phase-locking in deterministic dynamical systems, |*φ*_*n*,*m*_(*t*)| < *const* with *φ*_*n*,*m*_(*t*) = *nφ*_*b*_(*t*) − *mφ*_*s*_(*t*), does not hold in the presence of strong fluctuations because of the appearance of noise-induced phase slips^4^,^6^,^12^. The parameters *n* and *m* are integers, and *ϕ*_*b*,*s*_ are phases of the bundle and the stimulus, respectively, defined on the real line. If the phase-locking condition is fulfilled, *n* stimulus cycles should *always* fit in *m* bundle oscillations. In the presence of noise, however, entrainment can only be treated in a statistical fashion, as the appearance of preferred values in the distribution of the cyclic phase difference *ψ*_*n*,*m*_ = *φ*_*n*,*m*_ mod 2*π* (see SI. 2). To experimentally determine *ψ*_*n*,*m*_, the phase of the bundle *ϕ*_*b*_ was computed by performing the Hilbert transform on the bundle response^4^,^33^, and the phase of the stimulus *ϕ*_*s*_ was assumed to be 2*πf*_*s*_*t*.

The n:m vector strengths, defined as *R*_*n*,*m*_ = 1/*N*|∑_*j*_exp(*iψ*_*n*,*m*_(*t*_*j*_))|, provide quantitative measures of the contribution of the n:m modes of entrainment to the full hair bundle response. Here, *N* is the number of elements in the sum, and index *j* corresponds to time. A bundle response that is fully locked in a specific n:m mode would correspond to a constant value of *ψ*_*n*,*m*_ in time, and would therefore exhibit a vector strength *R*_*n*,*m*_ of 1. A random response of the bundle would exhibit a homogeneous distribution of *ψ*_*n*,*m*_ between 0 and 2*π*, resulting in a vector strength *R*_*n*,*m*_ of 0. We computed these vector strengths for signals applied at various amplitudes and frequencies. Since each stimulus protocol is applied for a limited amount of time (see Materials and Methods), one could obtain a spurious *R*_*n*,*m*_, even for an infinitely week stimulus applied at a frequency close to that of the spontaneous oscillation. Over the finite duration of the recording, the stochastic bundle phase diffusion is not sufficient to destroy the phase coherence between the spontaneous oscillation and a fictive stimulus. We evaluated this spurious component by applying the same analysis to bundle motility measured *without* an applied stimulus, and subtracted the result 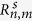 from *R*_*n*,*m*_: 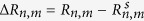. Consequently, Δ*R*_*n*,*m*_ reflects only the effect of the stimulus on the vector strength of the hair cell’s response.

**Figure 2:**
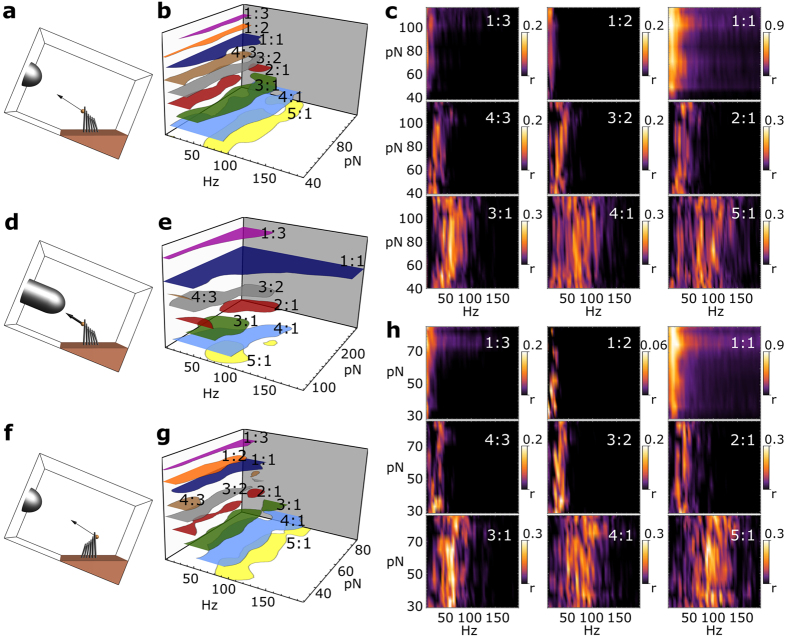
Multi-mode synchronization. Schematics of bundle stimulation (**a**) toward or (**f**) away from the tallest row of stereocilia. (**c**) and (**h**) present Δ*R*_*n*,*m*_ for various modes of synchronization. Rayleigh test was applied to Δ*R*_*n*,*m*_ with a 5% level of significance. Only vector strengths above the critical value r = 0.027 are displayed. (**d**) Schematics of high-amplitude bundle deflection toward the tallest row of stereocilia. The panels presenting the corresponding Δ*R*_*n*,*m*_ are shown in the SI. 3a. (**b**), (**e**), and (**g**) display overlaid regions of maximal n:m synchronization. The colored areas delimit the region where Δ*R*_*n*,*m*_ is larger than 35% of its maximum value.

The magnetic stimulus was applied so as to either pull the bundle toward (Fig. 2a) or away (Fig. 2f) from the tallest row of stereocilia. Figure 2c and h present the obtained Δ*R*_*n*,*m*_. A Rayleigh test was applied to Δ*R*_*n*,*m*_, and the plots display only the values that were found to be statistically significant. As can be seen from the figures, entrainment was observed for various orders of mode-locking, including fractional locking. High-order regimes of synchronization were consistently seen in the 12 cells studied.

Overlaid synchronization regions in Fig. 2b and g show significant overlaps, where the bundle oscillation could mode-lock to the imposed signal with different ratios of frequencies. Apart from the 1:1 mode, each Δ*R*_*n*,*m*_ remained lower than ~0.3, as multiple modes contributed jointly to the bundle response. Different synchronization modes contributed alternately to the bundle response. The respective n:m vector strength values indicate the contribution of each mode to the global bundle response, *integrated over time* during the full stimulus train. When the amplitude of stimulation was increased, 1:1 mode-locking was favored (Fig. 2d and e). Records obtained by pulling bundles toward (Fig. 2a–c) versus away (Fig. 2f–h) from the tallest row of stereocilia exhibited strikingly similar characteristics.

### Intermittent mode-locking

**Figure 3:**
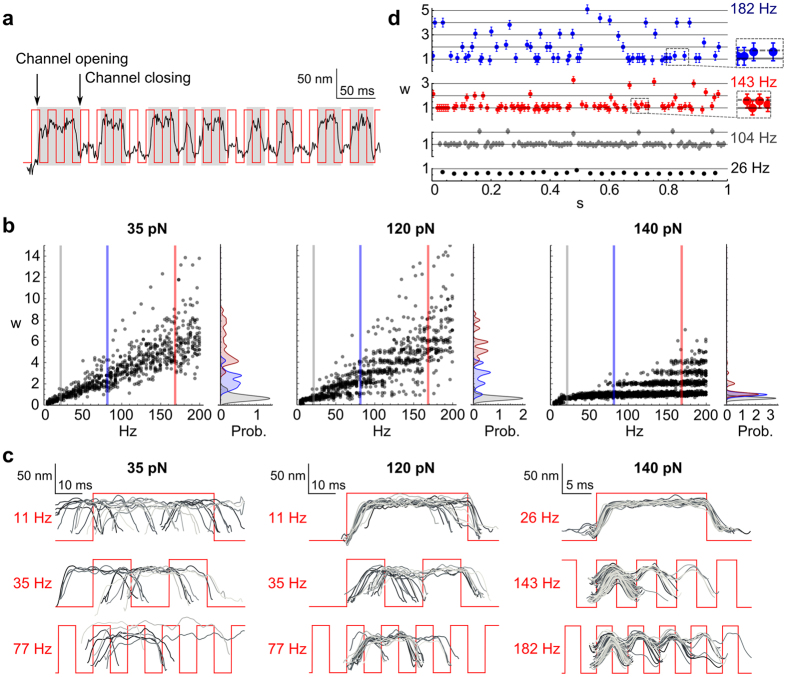
Intermittent mode-locking. (**a**) Detection of bundle oscillations. (**b**) Local winding number *w* as a function of the stimulus frequency, for increasing stimulation intensity. The dots are grey and semitransparent to distinguish isolated points from high density regions. The probability density function of *w* is presented for the three indicated stimulus frequencies. (**c**) Superposition of bundle oscillations for different stimulus intensities and frequencies. (**d**) Local winding number *w* as a function of time for a 140 pN stimulation, at different frequencies. Fractional mode-locking was observed and is shown in the expanded views (dashed boxes to the right of the plots). The dashed line in the expanded regions represents the 4:3 mode.

As shown in Fig. 2, there exist regimes of stimulation where the bundle can phase lock in different ratios to the applied frequency. We focused next on the dynamics of entrainment in these regimes of overlap. Each bundle oscillation, defined as the excursion between the opening (positive excursion) and subsequent closing (negative excursion) of the channels, was detected with software developed in Matlab^34^ (Fig. 3a). The *local winding number w* is defined to be the ratio of the period of an oscillation to the period of the stimulus. A sequence of 15 to 70 winding numbers was obtained for every stimulus condition.

Figure 3b plots the dependence of the winding number on the applied frequency, for selected stimulus amplitudes. No phase locking was detected at weak signals. The dispersion observed in the measured *w* originated from the innate width of the spectral density of spontaneous oscillations. At intermediate signals, bands appeared in the plots, indicating different modes of phase-locking. The overlap between the bands indicates that the system can phase lock to the applied signal at different ratios to the stimulus frequency. At higher amplitudes of stimulation, the bundle favored low winding numbers, and the bands sharpened.

To further characterize the overlap between bands of synchronization, we plotted the local winding number as a function of time, for different frequencies of stimulation (Fig. 3d). An intermittent flicker between different winding numbers was observed.

### Ensemble response to a broad range of frequencies

Due to the flicker between different n:m modes, prominent over a wide range of amplitudes, any individual hair bundle cannot unambiguously encode stimulus frequencies higher than *f*_0_. We next assessed how an ensemble of such bundles would respond to the same stimulus. Assuming the hair cell to be an ergodic system, we separated each record of the bundle response into sequential oscillations. The individual oscillations were superposed, preserving their phase relative to the stimulus (Fig. 3c). Each bundle oscillation occurred at a number of different modes of phase-locking to the signal. However, the oscillation of the full ensemble reflected the periodicity of the stimulus. Even at higher frequencies, where 1:1 mode-locking was completely lost, the ensemble successfully encoded the stimulus frequency.

### Numerical simulations of multi-mode locking

**Figure 4:**
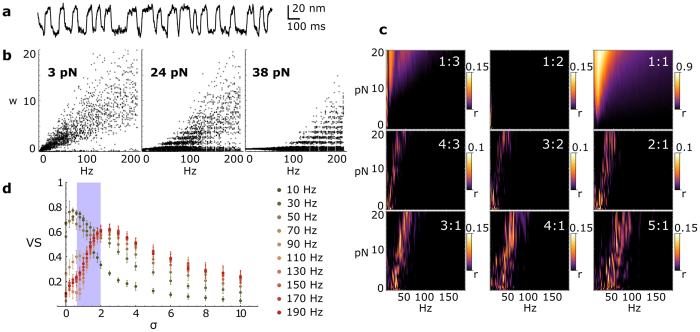
Simulations. (**a**) Simulation of spontaneous oscillations for *σ* = 0.8. (**b**) Local winding number w as a function of the stimulus frequency, for increasing stimulation intensity. (**c**) Δ*R*_*n*,*m*_ for various modes of synchronization. Rayleigh test was applied with a 5% level of significance. Only Δ*R*_*n*,*m*_ values above the critical value r = 0.019 are displayed. (**d**) Group Vector Strength VS as a function of noise intensity σ for a 10 pN stimulus, at frequencies from 10 Hz to 190 Hz. The group is composed of 20 bundles. Error bars are evaluated from 20 simulations. The gray region delimits the physiological noise.

To gain insight into the dynamical response of a single bundle as well as the behavior of an ensemble of identical bundles, we performed numerical simulations based on the models of active bundle motility developed in prior studies^15^,^26^,^35^. Following the description of stochastic bundle dynamics by Nadrowski *et al*.^35^, we modeled a single bundle with two variables that describe the deflection *X*(*t*) of the bundle and an intrinsic molecular motor displacement *X*_*a*_(*t*). In dimensionless form, the model reduces to:


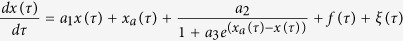



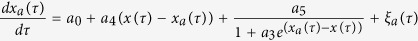


with *x*(*τ*) = *X*(*t*)/*δ, x*_*a*_(*τ*) = *X*_*a*_(*t*)/*δ, τ* = *t/τ*_0_, *δ* a characteristic length of the system, *τ*_0_ a characteristic time of the system, *f*(*τ*) the dimensionless stimulus, *ξ*(*τ*) and *ξ*_*a*_(*τ*) Gaussian white noise terms with standard deviation *σ*. The parameter values for *a*_*i*_ were adopted from Nadrowski *et al*.^35^, and defined in Materials and Methods; values were chosen that poise the system in the regime of limit cycle oscillation^35^,^36^,^37^. The natural frequency of a noiseless bundle was 6.5 ± 0.5 Hz. The bundle response was simulated under different stimulus conditions and was analyzed with the same procedures applied to the experimental data. The numerical model exhibited the same characteristic behavior for the local winding number (Fig. 4b), showing stochastic flicker between different modes. Further, simulations yielded high-order synchronization regimes with features similar to the experimental results (Fig. 4c). Using the model, we verified that a sinusoidal stimulus evokes the same characteristic response as a square wave stimulus (data not shown), indicating that our observations were not affected by the specific waveform of the periodic stimulus.

Numerical simulations allowed us to examine the role played by noise in the complex response of both a single bundle and an ensemble of bundles. In the case of a single bundle, as the noise intensity increased, we observed an evolution from sharp, non-overlapping Arnold Tongues with high vector strength values to broad, overlapping regions of synchronization, showing lower vector strength values (see SI. 3b–d). The model thus indicates that the coexistence of multiple synchronization modes is dependent upon the presence of a stochastic process.

Focusing on the response of an ensemble of bundles, we defined a *Group Vector Strength VS* that quantifies the ability of the ensemble to collectively encode the stimulus frequency (see SI. 4). The initial conditions for hair bundles within the group were randomized in the simulation. Increasing the noise intensity had a striking effect on *VS*. At low stimulus frequencies, *VS* decreased monotonically with noise. However, at high stimulus frequencies, *VS* showed a maximum at a non-zero noise intensity (Fig. 4d). The presence of noise therefore helps the ensemble to encode high frequencies, analogous to stochastic resonance predicted for individual hair bundles^38^. Extracting the intensity of physiological noise from our data, and comparing it to results of the simulations, we found that the innate biological noise is in the range that offers a compromise between the enhancement of detection of high frequencies and maintaining the detection of low frequencies (see SI. 5). Our estimates of the biological noise intensity (0.6 < σ < 2) are consistent with the values used in prior literature, converted into dimensionless form^35^,^39^.

## Discussion

Nonlinearity of the auditory system has been extensively demonstrated experimentally and modeled using dynamic systems theory^23^,^34^,^35^,^36^,^40^,^41^,^42^. Variation of internal control parameters poises hair cells near different bifurcations, leading to different phase-locking characteristics^13^,^36^,^38^. Hair bundle exhibits a regime of spontaneous limit cycle oscillation^15^, the frequency of which reveals an internal time scale defined by the active processes within the bundle. This time scale was seen to be slower than the response needed to explain the full range of frequency detection. We hence explored how a relatively slow nonlinear oscillator responds to frequencies that are high with respect to its characteristic frequency.

A common feature of nonlinear oscillators is that they can exhibit high-order synchronization^4^. Our results indicate that higher-order mode-locking can be robustly observed in hair cells of the inner ear. This behavior was found to form broad regions of entrainment, with significant overlaps, leading to regimes of multiple possible winding numbers. As a result, rather than displaying the traditional devil’s staircase structure^4^, the dependence of the winding number on the stimulus frequency showed overlapping plateaus, with intermittent flicker between them. Simulations indicated that this flicker is induced by noise in the response of individual hair bundles. How might such a stochastic system reliably detect higher frequencies of stimulation?

We propose that high frequencies are encoded in the collective response of an ensemble of bundles. Detection requires both the nonlinearity and the stochasticity of the system: the nonlinear response of an individual hair bundle enables it to phase-lock to a signal in different n:m modes, and the stochasticity enhances detection of high frequencies. At the level of a single hair bundle, the presence of noise increases the probability of transitions between channel-open and channel-closed states^38^. For strong stimuli, this effect favors high frequency synchronization (see SI. 5a). If the stimulus is weak, however, the noise destroys the phase information, and synchronization is lost. The major impact of noise arises from its effect on signal detection by an ensemble of hair cells. We showed that the response of an ensemble of noisy bundles, each flickering randomly between different n:m modes, enables a more efficient mapping of the stimulus periodicity than the response of an ensemble of deterministic bundles, having random initial conditions. This effect is preserved even under weak stimulus (see SI. 5b and c).

The model proposed here produces very weak frequency selectivity, as the oscillators phase-lock to a wide range of signals, at various winding numbers, thus producing a very sensitive broadband detector. The resulting behavior is consistent with the properties of the amphibian sacculus^29^, and other low-frequency vestibular and auditory systems, which display high sensitivity but broad tuning. Further, the notion that detection proceeds via an ensemble of hair bundles is consistent with the highly convergent patterns of innervation in the sacculus^29^. Signal detection beyond the hair bundle is outside the scope of the current work; however, experimental evidence supports phase-locking of stochastic spikes in the auditory neurons^43^,^44^. An analogous model has also been proposed for the response of an ensemble of neurons, as a means of encoding frequencies than exceed the firing rate of any of the individual neurons^45^,^46^. We demonstrate here the crucial role of the noisy flicker between different modes in achieving this effect.

A hair cell of the inner ear constitutes an example of a non-equilibrium system, whose nonlinear response is crucial for its biological purpose: extremely sensitive detection of sound. We observe high-order mode-locking between actively oscillating hair bundles and an imposed signal, with intermittent switching between different synchronization modes, originating from the combination of intrinsic noise and nonlinearity. We hypothesize that a collection of hair cells could take advantage of this effect to efficiently detect high frequencies of stimulation.

## Materials and Methods

### Biological preparation

The sacculi were excised from the inner ear of the North American bullfrog (*Rana catesbeiana*) and mounted over a 1 mm hole in a two-compartment experimental chamber, with hair cells exposed to artificial perilymph (110 mM Na^+^, 2 mM K^+^, 1.5 mM Ca^2+^, 118 mM Cl^−^, 3 mM D-glucose, 1 mM sodium pyruvate, 1 mM creatine, and 5 mM HEPES) on the basal side and endolymph (117.5 mM K^+^, 2 mM Na^+^, 0.25 mM Ca^2+^, 118 mM Cl^−^, 3 mM D-glucose, and 5 mM HEPES) on the apical side. The solutions were made to mimic the ionic conditions in the sacculus of an intact animal. The solutions were oxygenated for ~15 min prior to use. The overlying otolithic membrane was removed from the epithelium with an eyelash tool, following an 8 min enzymatic dissociation with 15 μg.mL^−1^ collagenase IV (Sigma-Aldrich), dissolved in artificial endolymph with 4 mM Ca^2+^. Spontaneous oscillation of hair bundles was typically observed for several hours after dissection. All animal-handling protocols were approved by the UCLA Chancellor’s Animal Research Committee (Protocol Number ARC 2006–043–13C), in accordance with federal and state regulations.

### Conjugation of magnetic particles to the hair bundle

Carboxylate-modified Sera-Mag Speedbeads (Fisher Scientific) were conjugated to the hair bundles via letin (Concanavalin A, conA) - glycoprotein interaction. After diluting 20 μL of the bead solution (50 mg/mL^−1^) in 1 mL of 10 mM phosphate buffer (pH 7.4), the beads were isolated by 2 min centrifugation at 1500 rcf and dispersed in 1 mL of phosphate buffer. EDC (383 μg) and sulfo-NHS (800 μg) were introduced into the solution to activate the surface of the magnetic beads, and dissolved for 15 min with gentle shaking. The beads were then isolated by centrifugation at 1500 rcf for 2 min and dispersed in 1 mL of phosphate buffer. 1.8 mg of conA type VI was dissolved in the solution and allowed to react with the beads for an hour under gentle shaking. When the reaction was finished, the beads were isolated by centrifugation at 1500 rcf for 2 min and dispersed in 1 mL of phosphate buffer; the rinse was repeated three times. After centrifugation of 100 μL of the resulting bead solution the magnetic beads were washed in 200 μL of endolymph and then dispersed in 100 μL of endolymph. One drop (~50 μL) of this bead solution was placed on top of the hair bundle preparation for 30 min. During the 30 min incubation, the two-compartment chamber was flipped upside down to avoid gravitational sedimentation of the beads on the surface of the sacculus.

### Detection of hair bundle motility

Experiments were conducted under an upright optical microscope (Olympus B51X) with a 60x water-immersion objective (LUMPLFLN60X, 1.0 NA) and illuminated with an EXFO X-cite 120 W light source. Images were further magnified by a 2x relay lens. They were recorded with a Hamamatsu Orca-Flash4.0 Scientific CMOS camera at 1000 frames per second. The camera was triggered by an analog output module from National Instruments (NI 9263), such that the beginning of the record coincided with the onset of the magnetic stimulus train. The observed bundle was oriented to oscillate along the horizontal axis. Its motion was tracked with software written in Matlab (The MathWorks), which calculates the center of mass of the hair bundle. Time-dependent traces of the movement were then obtained by plotting the center of mass for each frame of the recording. The noise levels in these recordings were estimated to be ~3–5 nm.

### Electromagnetic probe

The electromagnetic probe was constructed following previously described methods^30^,^47^. A copper wire coil, 70 Ω in resistance, was wound 1000 times around a permalloy magnetic probe tip (DSF System, Korea). A plastic capillary tube was used to create a water circulation jacket around the coil, to prevent overheating of the electromagnet. A permalloy 75 rod (Ni 75%, Fe 25%, 0.8 mm diameter, 25 mm length) was used as the electromagnet core material. The tip of the probe was 10 μm wide. Figure 1a illustrates the configuration of the experiment.

### Magnetic stimulation of a hair bundle

The voltage driving the electromagnet coil was generated by an analog output module from National Instrument (NI 9263), connected to an amplifier (PZ-150M, Burleigh), and the current in the coil was monitored by an analog input module from National Instrument (NI 9215). The waveform for the applied voltage was generated using LabVIEW software (National Instrument). The electromagnetic probe was mounted on a motorized micromanipulator (Siskiyou Inc.) and positioned close to the target hair bundle, which had been conjugated with 1–3 magnetic beads. The probe was placed either on the side of the tallest or the smallest row of stereocilia. The electromagnetic probe stimulated the bundle with square waves, sent at 66 frequencies ranging from 5 to 200 Hz in 3 Hz increments, and 14 intensities. The intensity range was dependent on the relative position of the bundle and the probe tip. The maximum force intensity (i.e. with the probe at magnetic saturation) was determined as describe above. The other intensities were calibrated by applying the complete experimental stimulus protocol to an elastic glass rod with magnetic beads attached. The deflection of the rod yielded the ratios between the applied force intensities. For each stimulus intensity, the frequencies were presented in a random order. The different intensities were also randomized to avoid any artifacts due to degradation of the bundle. Twenty stimulus cycles were presented for frequencies below 20 Hz, and one second of stimulation was applied for higher frequencies. After conclusion of each experiment, the bundles still exhibited spontaneous oscillation.

### Parameters used in the numerical model

We applied the model developed by Nadrowski *et al*.^35^ in dimensionless form


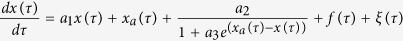



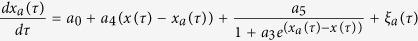


The parameters in the dimensionless form are related to those in ref. 35: 
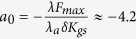
, 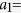 
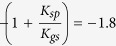
, 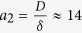, *a*_3_ = *A* ≈ 2.5 × 10^7^, 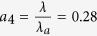, 
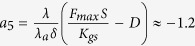
, *x*(*τ*) = *X*(*t*)/*δ, x*_*a*_(*τ*) = *X*_*a*_(*t*)/*δ, τ* = *t/τ*_0_, *τ*_0_ = *λ/K*_*gs*_ ≈ 3.7 × 10^−3^, *f*(*τ*) = *F*_*ext*_(*t*)/*δK*_*gs*_, *ξ*(*τ*) = *η*(*t*)/*δK*_*gs*_, *ξ*_*a*_(*τ*) = *η*_*a*_(*t*)/*δK*_*gs*_.

We applied the model in the limit where the calcium concentration relaxes instantaneously, and with *C*_0_ = 0. We adopted the parameter values chosen by Nadrowski *et al*.: *γ* = 0.14, *λ* = 2.8 μN.s.m^−1^, *λ*_*a*_ = 10 μN.s.m^−1^, *K*_*gs*_ = 750 μN.m^−1^, *K*_*sp*_ = 600 μN.m^−1^, *d* = 8.7 nm, *N*_*a*_ = 3000, *S* = 0.65, *f*_*max*_ = 352 pN, *N* = 50, *T* = 25 °C, Δ*G* = 10 *k*_*B*_*T*.

All the simulations and data analysis were performed using Wolfram Mathematica 10.

## Additional Information

**How to cite this article**: Levy, M. *et al*. High-order synchronization of hair cell bundles. *Sci. Rep.* **6**, 39116; doi: 10.1038/srep39116 (2016).

**Publisher's note:** Springer Nature remains neutral with regard to jurisdictional claims in published maps and institutional affiliations.

## Supplementary Material

Supplementary Information
