# Supplementary material for: High-order synchronization of hair cell bundles
Source: Sci Rep. 2016 Dec 15;6:39116. doi: 10.1038/srep39116 (PMC5156917; doi:10.1038/srep39116)
Supplement: Supplementary Information [file srep39116-s1.pdf]

Supplementary Information

High-order synchronization of hair cell bundles

Authors: Michael Levy[1], Adrian Molzon[1], Jae-Hyun Lee[2], Ji-wook Kim[2], Jinwoo Cheon[2] and Dolores Bozovic[1*]

[1] *Department of Physics and Astronomy, California NanoSystems Institute, University of California, Los Angeles, California 90095, United States*

[2] *Center for Nanomedicine, Institute for Basic Science (IBS), Seoul 03722, Republic of Korea*

Yonsei-IBS Institute, Yonsei University, Seoul 03722, Republic of Korea

Department of Chemistry, Yonsei University, Seoul 03722, Republic of Korea

Contact information: bozovic@physics.ucla.edu

Calibration of the 3D magnetic force

The magnetic field gradient $\vec{\nabla}B$, generated by the probe tip once it has reached magnetic saturation, was calibrated by tracking the velocity of magnetic beads dispersed in a solution containing 99% glycerol. The magnetic force was counterbalanced by the viscous force given by the Stokes law: $\mu\vec{\nabla}B = 3\pi\eta d\vec{v}$, where $\mu = 6.5 \times 10^{-14}$ A.m^2 is the magnetic moment of an individual magnetic bead, $\eta = 0.6389$ Pa.s is the viscosity of the 99% glycerol solution, d = 1.1 μm is the mean bead diameter, and $\vec{v}$ is the bead velocity. The trajectories of ~50 magnetic beads were recorded in the symmetry plane of the probe tip (blue plane in Fig. 1a) and analyzed with the particle-tracking algorithm developed by the MOSAIC Group. The experimental 2D field gradient in the symmetry plane of the probe tip was fitted by the field gradient for an array of magnetic dipoles (SI.1a). An array of four dipoles aligned in the direction of their magnetic moments was sufficient to model the experimental field in the region of interest (in the proximity of the tip). The size distribution of the magnetic beads caused a 30% uncertainty in the value of the force. The force in the full 3D space around the probe was deduced by assuming cylindrical symmetry.

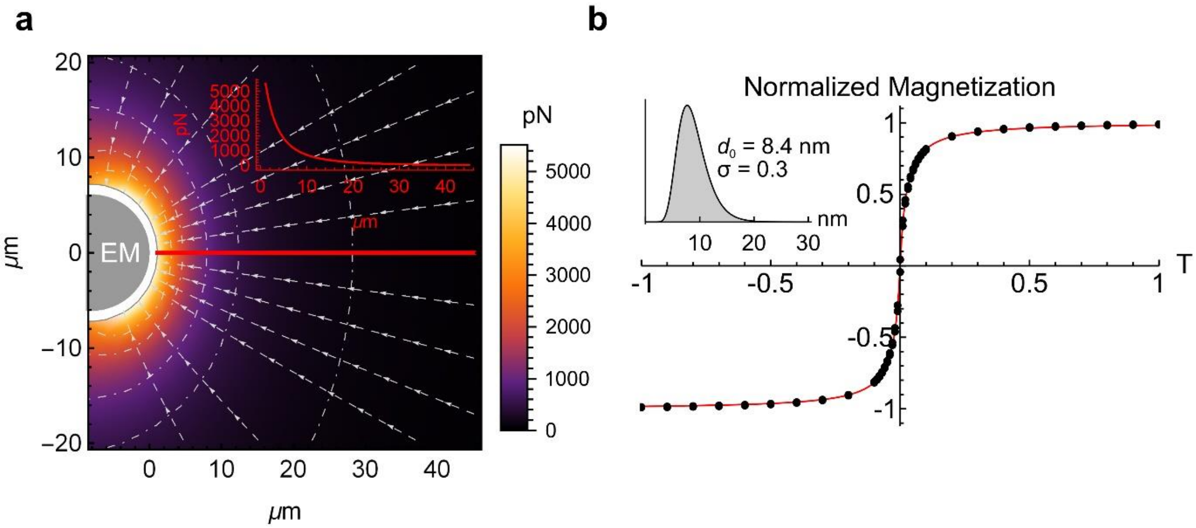

SI.1. Magnetic force and magnetization curve. *(a) Magnetic force acting on an average bead within the symmetry plane (blue plane in Fig. 1a). The origin of the plane corresponds to the tip of the electromagnet (EM). The inset present the force intensity along the red line. (b) Hysteresis curve of the beads (black points) and theoretical fit (red line). The size distribution of the magnetic nanoparticles embedded in the beads is presented in the inset.*

Magnetization measurement

The hysteresis curve of the beads was measured at room temperature (25˚C) with a superconducting quantum interference device (SQUID, Quantum Design MPMS7). No remnant magnetization was found, demonstrating the superparamagnetic state of the beads. The curve was well fit by weighting the Langevin expression $M = M_s(\coth\xi - 1/\xi)$ by a log-normal distribution of the nanoparticle diameter d, $P(d) = 1/(\sqrt{2\pi}\sigma d) \times \exp(-\ln^2\left(\frac{d}{d_0}\right)/2\sigma^2)$, with M_s the saturation magnetization of the magnetic material,

$\xi = \mu_0 M_s B\pi d^3/6k_B T$ the Langevin parameter, B the applied magnetic field, k_B the Boltzmann constant, T the temperature, σ the polydispersity index, and d_0 the characteristic diameter[1]. The two parameters of the nanoparticle size distribution were determined by fitting the model to the hysteresis curve (SI.1b): $d_0 = 8.4$ nm and $\sigma = 0.3$.

Determination of the phase of the bundle $\boldsymbol{\phi_b}$

The bundle position $X(t)$ was recorded as a function of time. In order to evaluate the phase of the bundle $\phi_b(t)$ we constructed the corresponding analytic signal $\zeta(t)$, a complex function of time defined as:

$$\zeta(t) = X(t) + i\tilde{X}(t) = A(t)e^{i\phi_b(t)}. \qquad 1$$

The function $\tilde{X}(t)$ is the Hilbert transform of $X(t)$

$$\tilde{X}(t) = \pi^{-1} P.V. \int_{-\infty}^{\infty} \frac{X(\tau)}{t-\tau} d\tau \qquad 2$$

where P.V. means that the integral is taken in the sense of the Cauchy principal value. The instantaneous amplitude $A(t)$ and the instantaneous phase $\phi_b(t)$ are uniquely defined.

Distribution of the cyclic phase difference $\psi_{n,m}$

Examples of hair bundle motility under stimulation at frequencies that were lower, comparable, or higher than the characteristic frequency f_0 of the bundle are shown in Fig. 1. SI.2. presents the corresponding $\psi_{n,m}$ distributions for different n and m values. In the case of a stimulus frequency close to f_0, a sharp peak appeared in the 1:1 histogram, and increased with increasing stimulus intensity. The other modes did not develop; the two symmetric peaks in the 2:1 histogram are artifacts of the 1:1 synchronization. When frequencies significantly lower or higher than f_0 were applied, sharp peaks or smooth anisotropies appeared in the histograms, in positions that are distinct from the 1:1 peak. These features increased with increasing stimulus intensity, and suggest the presence of high-order modes of phase-locking. Note that the peak in the 1:1 histogram shifted with increasing stimulus frequency. Bundle motility shifted from exhibiting a phase lead to a lag with respect to the phase of the applied stimulus, as observed in prior literature[2]. The phase lead was shown to result from the presence of an active process in the bundle.

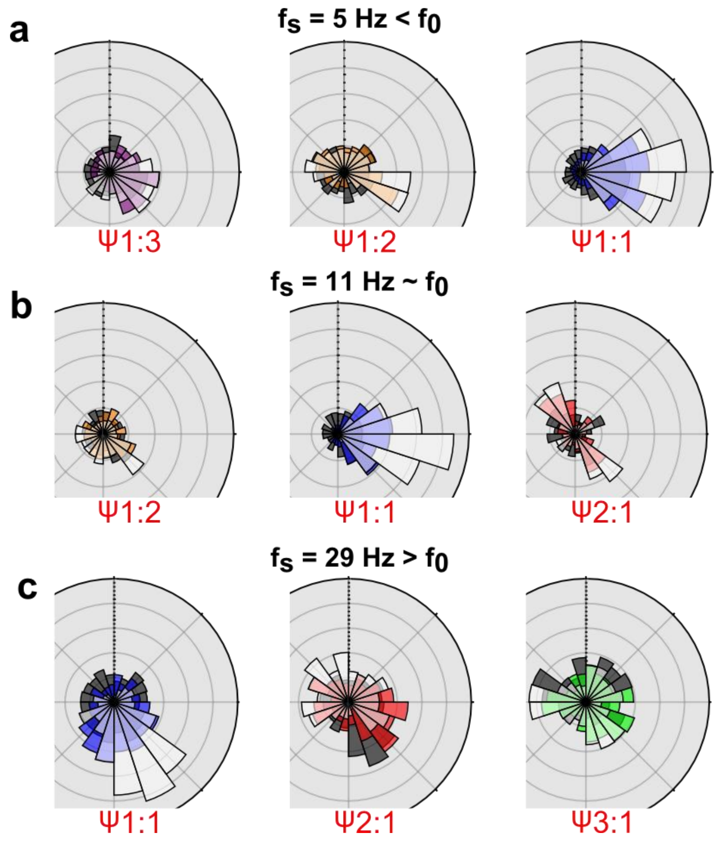

SI.2. Histogram of $\psi_{n,m}$. *Distributions of the cyclic phase differences $\psi_{n,m}$ for hair bundle oscillations in the presence of a square-wave stimulus with frequency f_s, applied at increasing intensities for (a) $f_s < f_0$, (b) $f_s \sim f_0$, and (c) $f_s > f_0$. The distributions that are shown in dark gray, colors, and light gray correspond to the smallest (43 pN), medium (77 pN), and largest (112 pN) stimulus intensity, respectively. Every distribution passes the Rayleigh test at a 5% significance level except the one for $\psi_{1,2}$ corresponding to a 43pN stimulus at 5Hz.*

High-amplitude bundle stimulation toward the tallest row of stereocilia:

Experiments and Simulations

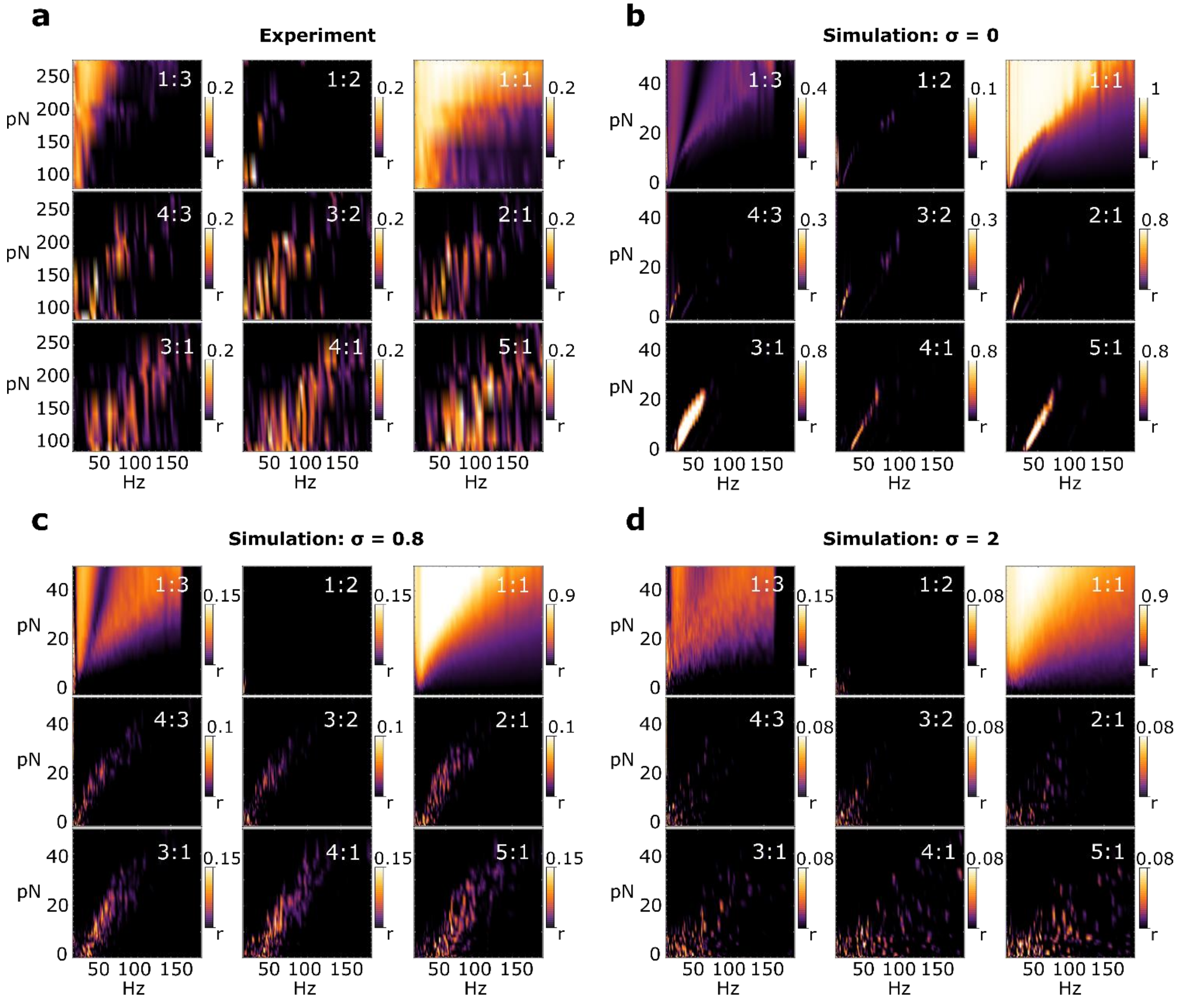

SI.3. Strong bundle stimulation: experiment and simulations. *(a) Panels presenting experimental measurements of* $\Delta R_{n,m}$ *for various modes of synchronization, with the bundle subjected to strong stimulation (see Fig. 3d and 3e). Rayleigh test was applied to*

$\Delta R_{n,m}$ with a 5% level of significance. Only vector strengths above the critical value r = 0.039 are displayed. As the stimulus amplitude was increased, the 1:1 mode-locking was favored. Panels (b), (c), and (d) present simulated values of $\Delta R_{n,m}$ for various modes of synchronization; bundles were simulated with $\sigma = 0$, $\sigma = 0.8$ and $\sigma = 2$ respectively. A graphical smoothing technique consisting of averaging over the closest neighbors was applied. Rayleigh test was also applied to $\Delta R_{n,m}$ with a 5% level of significance. Only vector strengths above the critical value r = 0.019 are displayed. The critical value r depends on the number of measurements. It is different between the experiment and the simulation. Note that the force intensities for simulations differ from the experimental results; parameters obtained from Nadrowski et al [3] were not adjusted to fit the data, but used to capture the characteristic behavior.

Simulation of the response of an ensemble of hair bundles

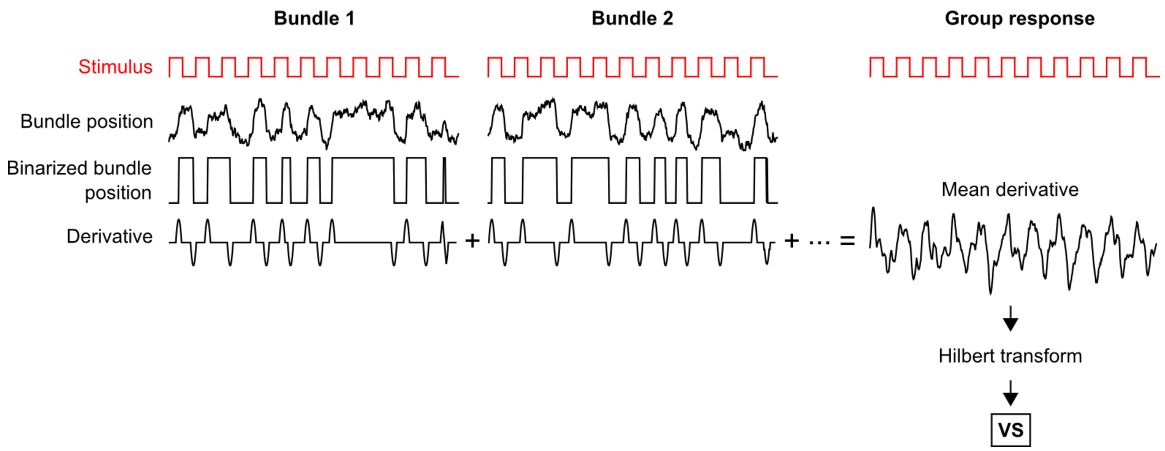

SI.4. Calculation of the group response VS. *The response of an ensemble of bundles was simulated numerically. For each bundle, the position was fit to a series of square waves of various duration; the derivative was calculated from the fit. The vector strength VS of the mean of all the derivatives was computed by performing the Hilbert transform.*

Simulations of the response of an ensemble of bundles

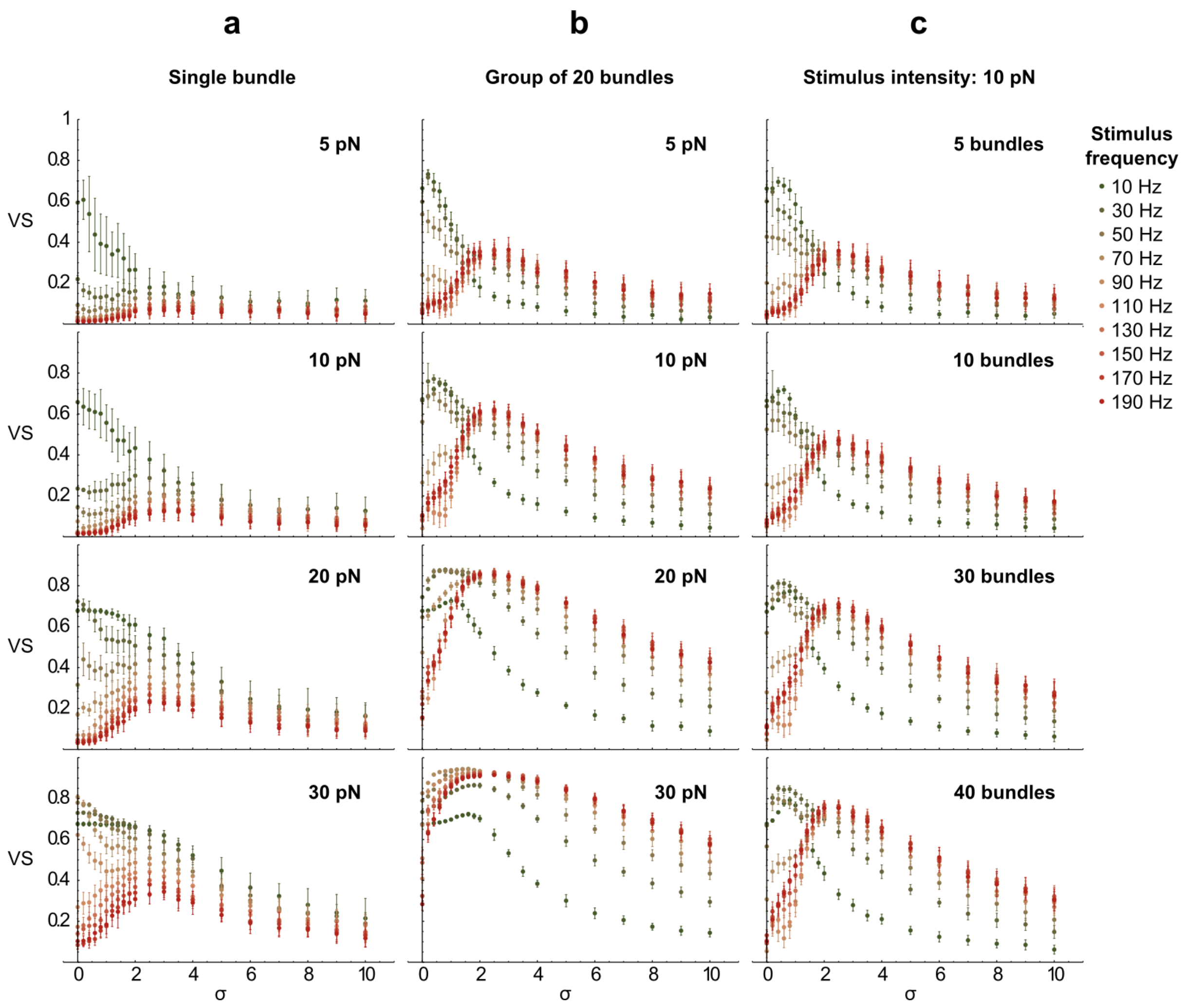

SI.5. Simulated response of an ensemble of bundles: VS(σ). *(a) Effect of the stimulus intensity on the response of a single bundle. (b) Effect of the stimulus intensity on the response of a group of 20 bundles. (c) Effect of the number of bundles in the ensemble. The stimulus intensity was fixed at 10 pN. Error bars were evaluated from 20 simulations.*

Physiological noise intensity

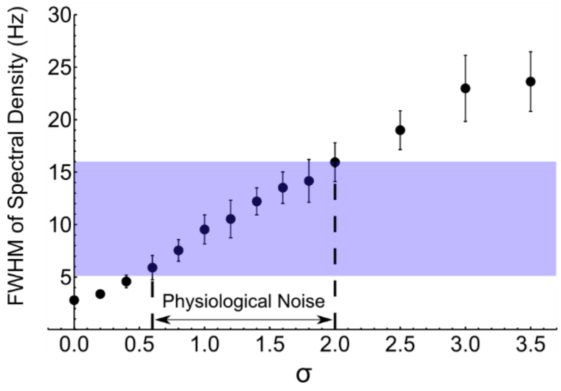

SI.6. Evaluation of the physiological noise. *The spectral density of spontaneous bundle oscillations was simulated for different noise intensities. The Full Width at Half Maximum (FWHM) was evaluated as a function of the noise intensity σ. The region in gray delimits the experimental FWHM values. Error bars were evaluated from 20 simulations.*

References

1. Levy, M. *et al.* Correlating magneto-structural properties to hyperthermia performance of highly monodisperse iron oxide nanoparticles prepared by a seeded-growth route. *Chemistry of Materials* **23,** 4170–4180 (2011).

2. Martin, P. & Hudspeth, A. J. Active hair-bundle movements can amplify a hair cell's response to oscillatory mechanical stimuli. *Proc. Natl. Acad. Sci. U.S.A.* **96,** 14306–11 (1999).

3. Nadrowski, B., Martin, P. & Jülicher, F. Active hair-bundle motility harnesses noise to operate near an optimum of mechanosensitivity. *Proceedings of the National Academy of Sciences of the United States of America* **101,** 12195–12200 (2004).
